# A Mismatch Between Patient Education Materials About Sickle Cell Disease and the Literacy Level of Their Intended Audience

**DOI:** 10.5888/pcd13.150478

**Published:** 2016-05-12

**Authors:** Elizabeth McClure, Jared Ng, Kelly Vitzthum, Rima Rudd

**Affiliations:** Author Affiliations: Jared Ng, Kelly Vitzthum, Rima Rudd, Harvard TH Chan School of Public Health, Department of Social and Behavioral Sciences, Boston, Massachusetts. Ms. McClure is also affiliated with Harvard TH Chan School of Public Health, Department of Social and Behavioral Sciences.

## Abstract

**Introduction:**

Despite the first goal of the 2010 National Action Plan to Improve Health Literacy, the literacy demands of much health information exceeds the reading skills of most US adults. The objective of this study was to assess the health literacy level of publicly available patient education materials for people with sickle cell disease (SCD).

**Methods:**

We used 5 validated tools to evaluate 9 print and 4 online patient education materials: the simple measure of gobbledygook (SMOG) to assess reading grade level, the Peter Mosenthal and Irwin Kirsch readability formula (PMOSE/IKIRSCH) to assess structure and density, the Patient Education Materials Assessment Tool (PEMAT) to assess actionability (how well readers will know what to do after reading the material) and understandability, the Centers for Disease Control and Prevention’s (CDC’s) Clear Communication Index (Index) to obtain a comprehensive literacy demand score, and the Printed Cancer Education Materials for African Americans Cultural Sensitivity Assessment Tool.

**Results:**

Materials’ scores reflected high reading levels ranging from 8th grade to 12th grade, appropriate (low) structural demand, and low actionability relative to understandability. CDC suggests that an appropriate Index score should fall in or above the 90th percentile. The scores yielded by materials evaluated in this assessment ranged from the 44th to the 76th percentiles. Eight of the 13 materials scored within the acceptable range for cultural sensitivity.

**Conclusion:**

Reading levels of available patient education materials exceed the documented average literacy level of the US adult population. Health literacy demands should be a key consideration in the revision and development of patient education materials for people with SCD.

## Introduction

Public health’s population-based strategies for improving community health include outreach to and communication with vulnerable populations. Patients, their families, and their communities need clear, understandable information; therefore, clear communication is a component of public health’s mission at the national, regional, state, and local levels (1). As indicated by the 2010 National Action Plan to Improve Health Literacy, accessible health information is key to promoting population health (2).

The first goal of the 2010 National Action Plan to Improve Health Literacy calls for the development and dissemination of health and safety information that is accurate, accessible, and actionable (2). However, surveys conducted by the US Department of Education and by the Organisation for Economic Co-operation and Development (OECD) indicate that large proportions of adults in the United States and in most industrialized nations have difficulty understanding commonly available written information (3–8). The most recent assessment of adult literacy skills indicates that more than half of US adults have difficulty using print materials and basic arithmetic in everyday activities and tasks (7). Each wave of literacy assessments of US adults indicated that minority population groups were more likely than majority population groups to have limited literacy skills (4,7,8). Approximately 80% of people with sickle cell disease (SCD) in the United States identify as black (9). The 2003 National Assessment of Adult Literacy (NAAL) found that only 2% of US black adults were proficient in general literacy skills compared with 13% of the general population (8). The 2006 NAAL subreport, which addresses health literacy, found that 12% of US adults were proficient in prose, document, and numeric health compared with only 2% of black adults (5). Although easily understandable health information should be accessible to everyone, special consideration should be given to making health information accessible to population groups with documented low literacy skills — those living in poverty and in under-resourced areas, members of minority population groups, and members of immigrant populations.

Unfortunately, as more than 2,000 peer reviewed studies showed, health information is often inaccessible because materials are written at reading levels that exceed the literacy skills of most US adults (3). Furthermore, one study in health literacy indicated that people with limited literacy were more likely to experience diminished health outcomes (10). The mismatch between the literacy skills of patients and the literacy demands of health education materials and instructions may play a significant role in enabling or inhibiting people to make healthful choices (6,11,12).

Insights from health literacy studies are directly applicable to public health’s mission to improve the health of communities and the prevention and management of chronic diseases. SCD is a significant concern among the many issues addressed in public health practice. This disease is disproportionately experienced by people of African, Mediterranean, or Latin descent (13) and affects an estimated 90,000 to 100,000 people in the United States (9,13,14). Several acute and chronic complications are associated with SCD, requiring complex disease management in both home and clinical settings. However, SCD patients in the United States have notably diminished comprehensive care services available to them, relative to other genetic disorders (15). Thus, patients with SCD and their family members could benefit from having appropriate educational materials about treatment options and procedures to help them in planning and making decisions (3). However, studies and investigations related to health literacy and SCD are absent from the literature. This study examines the literacy level required for use of available SCD educational materials (literacy demand) and the cultural appropriateness of such information for the intended audience.

## Methods

The lead author (E.M.) conducted an initial search of the literature to find widely available educational materials related to SCD. Because state health departments, public hospitals, and other public institutions frequently rely on free print and electronic information provided by national organizations when choosing educational materials for families and communities, we evaluated only free materials. We identified materials through the PubMed database by using the MeSH term “sickle cell anemia” or one of the key terms, “sickle cell” or “sickling,” in the title and abstract and the publication type, “patient education.” The educational materials were reviewed and were excluded if they were not written in English, if they were not free, if they were not intended for use in the United States, or if they did not meet the topic criterion of patient education. These criteria yielded 9 print and 4 online materials, which we then reviewed for literacy and numeracy demand (ie, the level of literacy and facility with arithmetic required for readers to understand the material and take appropriate action based on what they read).

We analyzed the educational materials by using several tools and guidelines to assess the characteristics of the materials (Table 1). The simple measure of gobbledygook (SMOG) readability test was used to ascertain school grade reading level. SMOG determines grade level with attention to both word and sentence length (16). The PMOSE/IKIRSCH measure (named for the individuals who developed the measure, Peter B Mosenthal and Irwin S. Kirsch) was used to assess structure and density in displays (lists, charts, graphs) contained in the materials (17). PMOSE/IKIRSCH provides a grade-level score based on the complexity of documents and the presence of all needed information within the confines of the document. A series of bullet points was considered a list if the content of each bullet point was less than a complete sentence.

**Table 1:** Comparison of Characteristics Captured by Four Tools for Assessing Health Literacy Demand

Literacy Assessment Tool			
SMOG	PMOSE/IKIRSCH	PEMAT	Index
**Organization**			
Tool does not assess this component	Simple-list structure	Material breaks or “chunks” information into short sections	Material uses bulleted or numbered lists
		Sections have informative headers	Material is organized in chunks with headings
	Combined-list structure (includes pie-charts and time-lines)	Presents information in logical sequence	Most important information is summarized in first paragraph or section
	Intersected-list structure (includes bar charts, line graphs, and maps)	Provides a summary	
	Nested-list structure (includes bar charts and line graphs with tested tables	Material uses visual cues	
	Material contains a reasonable number of labels	Material uses visual aids	
	Material contains a reasonable number of items	Visual aids reinforce content rather than distract	
	Dependence (material) does not make reference to information in an outside document)	Visual aids have clear titles or captions	
		Material uses illustrations and photographs that are clear and uncluttered	
		Material uses simple tables with short and clear row/column headings	
**Content of Main Message**			
Tool does not assess this component	Tool does not assess this component	Purpose is evident	Material contains one main message
		No distractors	Main message is at the top, beginning, or front
			Main message is emphasized with a visual cue
			Material contains visual(s) that convey or support the main message
			Material explains what is known or not known about topic
**Word Choice and Style**			
Material contains minimal necessary word length	Tool does not assess this component	Material uses common, everyday language	Material always uses language the primary audience would use
Material contains minimal necessary sentence length		Medical terms are defined	Main message and calls to action use active voice
		Active voice is used	
**Use of Numbers**			
Tool does not assess this component	Tool does not assess this component	Numbers are clear and easy to understand	Material always explains what the numbers mean
		Material does not expect user to perform calculations	Audience does not have to conduct mathematical calculations
**Risk**			
Tool does not assess this component	Tool does not assess this component	Tool does not assess this component	Material explains the nature of risk
			Material explains the risks and benefits of recommended behaviors
			If numeric probability is included, it is explained with text or a visual
**Actionability**			
Tool does not assess this component	Tool does not assess this component	Material states at least one action reader can take	Material includes one or more calls to action for primary audience
		Material addresses user directly when describing action	Material includes one or more behavioral recommendations for primary audience
		Action is broken down into manageable, explicit steps	Material explains why recommendation is important
		Material provides a tool that can help user take action	Material includes specific directions about how to perform the behavior
		Material explains how to use the charts, graphs, tables, or diagrams to take actions	

Abbreviations: Index, Centers for Disease Control and Prevention Clear Communication Index; PEMAT, Patient Education Materials Assessment Tool; PMOSE/IKIRSCH, [measure developed by Peter B. Mosenthal and Irwin S. Kirsch]; SMOG, simplified measure of gobbledygook.

Source: Christine E. Prue, PhD, MSPH, Associate Director for Behavioral Science, Centers for Disease Control and Prevention, National Center for Emerging and Zoonotic Infectious Diseases.

We used the Patient Education Materials Assessment Tool (PEMAT) to evaluate the understandability and actionability of materials (18).*Understandability* is the degree to which people with low health literacy can interpret key messages, and *actionability* is the degree to which people can know the proper next steps (eg, when and where to seek care, which healthful behaviors to adopt) on the basis of the information provided (19,20). In addition, the Centers for Disease Control and Prevention’s (CDC’s) Clear Communication Index (Index) was used to provide one overall outcome measure of readability and audience appropriateness, which is an amalgamation of measures related to the relevance of 1 to 4 factors — core items, behavioral recommendations, numbers, and risk (21). Because minority racial/ethnic groups make up most of the SCD patient population, the Index stresses that analyses and discourse must examine issues of culture in addition to, and in the context of, health literacy. The Index assesses appropriateness for the audience and, therefore, cultural appropriateness. However, to more specifically assess the materials’ cultural appropriateness, we also applied the Printed Cancer Education Materials for African Americans Cultural Sensitivity Assessment Tool (AACSAT). This tool measures acceptability in the cultural domain that the other measurement tools we used do not address, in the format, visual message, and written message (22).

Three authors (E.M., J.N., K.V.) independently scored all materials with each tool. As part of a training in health literacy theory and reading level measurement in a graduate course at the Harvard TH Chan School of Public Health, each of these 3 authors reviewed a set of materials unrelated to the subject matter and presented their findings to a panel of 8 experts trained in health literacy theory to analyze any differences, resolve errors, and establish consensus. Thereafter, each of the study materials was read and assessed by each of the 3 authors independently, and inconsistencies were resolved in group meetings of all authors. The PEMAT, Index, and AACSAT components each contain value judgments bounded by standardized parameters. For these assessments, the average of the reviewer scores was reported. When the 2 initial scores differed by more than 10%, the scores were discussed and recalculated by all 3 authors. The lead author (E.M.) was solely responsible for the AACSAT.

## Results

The PubMed search returned 6 sources for patient education information of which 3 sources were eliminated during initial review because they were not written in English, were not available free of charge, were not intended for use in the United States, or were not related to patient education. The 3 remaining sources of patient education materials were CDC (23), the National Institutes of Health (NIH) (24), and the American Academy of Family Physicians (AAFP) (25). NIH and AAFP materials were Web-based, and the CDC materials were formatted for print distribution but were available online. The NIH publication was a multipage site with comprehensive outlines of risks, diagnoses, symptoms, and treatments (24). It included lists, charts, statistical graphs, and other graphics. AAFP, however, provided a 1-page synopsis of SCD for parents of children with a new diagnosis of SCD. Its website contained no charts, statistical graphs, or other graphics (25). Five of the 9 materials from CDC addressed self-management and complication prevention. Those 5 CDC materials ranged in length from 1 to 40 pages of text, and 2 of the 5 included photo images. Four of the 9 materials focused on disease overview and disease inheritance. They ranged in length from 1 to 2 pages, and they all contained photos. Two of them contained an image illustrating the inheritance pattern of SCD (23). Table 2 presents the scores from each of the 5 tools for each of the educational materials.

**Table 2:** Health Literacy Demand Scores of 13 Patient Education Materials on Sickle Cell Disease, Evaluated by Measurement Tool

Measurement Tool							
Educational Material	SMOG^a^	PMOSE/ IKIRSCH Measure^b^	PEMAT Usability^c^	PEMAT Actionability^c^	PEMAT Overall^c^	Index^d^	AACSAT^e^
Toolkit for Living Well With Sickle Cell Disease (16)	10	5	68	58	64	57	3.0
Tips Sheet: Supporting Students with Sickle Cell Disease (16)	12	6	65	76	70	58	2.2
Fact Sheet: Sickle Cell Disease (16)	11	—f	74	0	38	44	3.2
Fact Sheet: Sickle Cell Disease and College (16)	11	—f	73	40	61	75	2.3
Fact Sheet: Sickle Cell and Pregnancy (16)	11	—f	68	36	52	55	3.0
Fact Sheet: Sickle Cell Trait	10	—f	75	27	51	63	3.1
Living Well With Sickle Cell Disease (16)	10	—f	70	28	49	59	3.0
Five Tips to Prevent Infection	10	—f	85	63	74	76	3.0
Emergency Guide: When to See the Doctor (16)	9	—f	83	64	72	64	3.2
NIH Web pages: What Is Sickle Cell Anemia (17)?	10	—f	78	0	42	46	2.5
NIH Web pages: Causes (17)	10	8	66	0	35	53	2.5
NIH Web pages: How Is Sickle Cell Anemia Treated (17)?	12	—f	44	18	32	52	2.0
AAFP Web page: When Your Child Has Sickle Cell Disease (18)	8	—f	75	62	68	71	2.9

Abbreviations: AACSAT, African Americans Cultural Sensitivity Assessment Tool; AAFP, American Academy of Family Physicians; Index, Centers for Disease Control and Prevention Clear Communication Index; NIH, National Institutes of Health; PEMAT, patient education materials assessment tool; PMOSE/IKIRSCH, [measure developed by] Peter B. Mosenthal and Irwin S. Kirsch; SMOG, simplified measure of gobbledygook.

a Numeric score indicating school grade reading level.

b Numeric score and ranking (very low = 3–5, low = 6-8, moderate = 9–11, high = 12–14, very high = 15–17); tool applies only to materials without lists or charts.

c Numeric component score and overall score (out of 100 possible points). PEMAT assesses usability (the degree to which people from different backgrounds can understand the messages conveyed) and actionability (the degree to which people from different backgrounds know what to do with the information [eg, when and where to seek care, which healthful behaviors to adopt] on the basis of the information provided).

d Percentile score out of 100 possible points (minimum score of 90 is considered passing).

e Numeric score out of 5 possible points (minimum score of 2.5 is considered acceptable).

f Contains no lists, charts, or graphs.

The SMOG scores of the materials we evaluated ranged from 8th grade to 12th grade reading level; most materials fell in the 10th grade to 12th grade range. A SMOG score of 7 or below is recommended for average readers. A score of 8 is generally assumed to represent the reading skills of average high school students (16). SMOG focuses on word and sentence length. Several sections of the assessed materials contained long complex sentences exceeding 3 lines of text.

Only 3 of the materials (printed or Web-based) contained lists, and none contained charts or statistical graphs. PMOSE/IKIRSCH assesses charts, statistical graphs, lists, and layout and is scored on a 17-point scale. All materials with lists received a PMOSE/IKIRSCH score indicating a low complexity level (Table 2). Scores in this range are estimated to require an 8th-grade literacy level. As indicated by the PMOSE/IKIRSCH tool, use of simple lists with 1 heading and a limited number of items minimizes literacy demand (17).

Next, all 13 materials were scored with PEMAT. All materials scored above the 50th percentile in understandability, except the “How Is Sickle Cell Anemia Treated” NIH Web page (Table 2). As noted, PEMAT evaluates understandability of materials (ie, patients from different backgrounds can understand the messages conveyed) and actionability (ie, patients from different backgrounds know what to do with the information), and scoring is based on clarity of purpose, organization, and difficulty of content (including numerical demand). PEMAT has no recommended cutoff for acceptable scores. Rather, the scores are meant to be used as relative indicators of quality when choosing between materials (18). Low PEMAT scores indicate that the materials assessed would be improved by simplifying content. For example, all numerical concepts should be presented in a way that does not require the reader to perform any calculations (19). Finally, all 13 materials scored low on PEMAT in actionability, relative to usability. Many of the materials discussed healthful behaviors but did not provide explicit instruction or diagrams demonstrating actions to be taken.

The Index yields a percentile score, and CDC states that documents with appropriate health literacy demand will score at the 90th percentile or higher (21,26). None of the materials received a score in the 90th percentile or higher, and some were below the 50th percentile (Table 2). The Index has 7 main areas of interest, including 2 behavior- or action-oriented foci and 2 numeric concept-related foci. Similar to the recommendations drawn from PEMAT, low scores from the Index assessment indicate that the included materials could be improved with more behavioral and instructive language.

Finally, the AACSAT printed cancer education materials yielded scores in the acceptable range for 8 of the 13 materials evaluated (Table 2). Scores were acceptable if materials achieved cultural relevance and appropriateness for the intended audience in format, content, and graphics (22). Acceptable scores from the AACSAT were obtained by meeting the audience-specific literacy demand requirements addressed in SMOG, PMOSE/IKIRSCH, PEMAT, and Index and inclusion of culturally relevant and modern imagery (22).

## Discussion

The free patient education materials assessed are those derived from a PubMed search meant to capture documents that clinicians would be likely to recommend to patients, that came from government agencies or professional societies, and that public health departments are likely to disseminate through community organizations and clinics. The reading level of the materials assessed fell between 8th-grade and 12th-grade levels, and these scores are considered too high for the general US adult population (27). Problematic content associated with high scores can be mitigated with attention to vocabulary (ie, substitute short and common terms for long, unusual, or technical terms. For example, use *doctor* instead of *physician*) and with attention to sentence length (ie, long sentences increase reading difficulty). The various charts and lists included in the assessed materials yielded literacy demand scores at appropriate levels. Scores related to the graphics explaining heredity of SCD, however, indicated high literacy and numeracy demand. Results also indicated a need for action-oriented language (eg, “Do X. Then do Y.” rather than something like “It is recommended that patients do X followed by Y.”) and instructions about what to do as a result of reading the materials. At the same time, findings indicated that some basic concepts were clearly described and that many of the materials contained culturally appropriate content.

Given the proportion of Americans proficient in quantitative literacy reported in the 2003 NAAL report and in numeracy reported in the 2013 OECD report, use of numeric content must be treated with caution (8). Broad assessments of the 13 materials evaluated suggest that the presentation of numeracy content was overly complex and lacked adequate explanation. The Index tool points out that an explanation with graphics is particularly important for mathematically complex concepts such as those displayed in the “inheritance tree” in patient materials related to risk and sickle cell trait (23–25). Insights at this level of detail demonstrate the strength of the Index, a more complex tool. The inheritance graphics and explanations of illustrations in educational materials for people with SCD require careful attention to ensure they are understandable by their intended audience.

Our assessment of the 13 patient education materials included in our analysis suggests that literacy and numeracy demands associated with use of these materials are likely to exacerbate difficulties people with SCD have in understanding patient education information designed to assist them in managing their disease. This may contribute to health literacy disparities and related health outcomes outlined by the National Action Plan to Improve Health Literacy (2). The educational materials addressing SCD receive scores that exceed recommended reading levels to ensure accessibility for the average American adult who has completed high school (27).

The content of the materials evaluated addressed some of the major issues related to SCD. Many issues that affect lifelong health of people with SCD, however, were not covered. Several patients lose primary and regular health care during the transition from pediatric to adult care (15). These ideas were covered in the publication “9 Steps to Living Well with Sickle Cell Disease in College,” but transition of care is not directly addressed in that publication (23). Additionally, management of SCD differs from childhood to adulthood, and no specific guidelines were offered for each stage of life in the materials assessed. Finally, perhaps the most complex issues in living with SCD relate to accessing information on pain management and routine care (28). The materials examined in this study did not address these issues directly.

Our study had 2 limitations. First, no patient group or review team apart from the authors was involved in the analysis of materials. Therefore, assessments were inadequate for drawing full conclusions regarding cultural relevance and appropriateness (29). However, AACSAT provided one indication of appropriateness of the patient education materials for their intended audience (22). Second, the sample was limited to free publications. Many educational materials for patients with SCD were developed by nongovernment organizations and by private institutions and were not necessarily free. Only free materials were included in this evaluation. Despite these limitations, this study provides an assessment of available materials through use of several different tools for assessing health literacy demand. Use of 5 different tools highlighted strengths and weaknesses of patient education materials in several domains of health literacy — organization, content, word choice, numeracy, usability, actionability, and cultural sensitivity.

Resulting scores for the 13 publications evaluated fell short of the standards articulated in the commonly referenced Doak, Doak, and Root text, *Teaching Patients with Low Literacy Skills*, which covers educational theories and applies them to improving health communication (27), and in the 2010 National Action Plan to Improve Health Literacy (2). A better match between the literacy requirements of the materials and the known literacy levels of their audience would better address information access needs of patients with SCD. Matching materials to their audience would improve the ability of people with SCD to make decisions and take healthful action.

The results of both the preliminary literature search and materials assessments suggest that there is a shortage of available and appropriate published information for people with SCD. Study findings indicate that SCD patient education materials should be revised or developed to use language tailored to their intended audience (24). In addition, input from patient focus groups to address appropriateness and usefulness (29) is critical.

This study supports the importance of health literacy as a key consideration in the development and revision of patient education materials for people with SCD. Health departments should assess the suitability of materials they distribute in their communities. In so doing, health departments can use existing tools such as the Index and related documentation for analyses. An understanding of the literacy skills of US adults must help shape the development of important health information (30). An awareness of the existing mismatch between commonly available health materials and the literacy levels of the intended audience can help inform strategic decisions of public health professionals for dissemination of information.

## References

[1] Frieden TR . Six components necessary for effective public health program implementation. Am J Public Health 2014;104(1):17–22. 10.2105/AJPH.2013.301608 24228653 PMC3910052

[2] National Action Plan to Improve Health Literacy. Washington (DC): US Department of Health and Human Services, Office of Disease Prevention and Health Promotion; 2010.

[3] Rudd RE . Health literacy skills of US adults. Am J Health Behav 2007;31(Suppl 1):S8–18. 10.5993/AJHB.31.s1.3 17931141

[4] Kirsch IS , Jungeblut A , Jenkins L , Kolstad A . Adult literacy in America: a first look at the findings of the National Adult Literacy Survey. Washington (DC): US Department of Education; 1993.

[5] Kutner M , Greenberg E , Jin Y , Paulsen C . The health literacy of America’s adults: results from the 2003 National Assessment of Adult Literacy (NCES 2006-483). Washington (DC): US Department of Education, National Center for Education Statistics; 2006.

[6] Lemke M , Gonzales P . Student and adult performance on international assessments of educational achievement. Washington (DC): US Department of Education, National Center for Education Statistics; 2006.

[7] Organisation for Economic Co-operation and Development. OECD skills outlook 2013: first results from the Survey of Adult Skills. Paris (FR): OECD Publishing; 2013. 10.1787/9789264204256-en

[8] White S , Dillow S . 2003 National assessment of adult literacy (NCES 2006-470). Washington (DC): US Department of Education, National Center for Education Statistics; 2005.

[9] Hassell KL . Population estimates of sickle cell disease in the U.S. Am J Prev Med 2010;38(4, Suppl):S512–21. 10.1016/j.amepre.2009.12.022 20331952

[10] Berkman ND , Sheridan SL , Donahue KE , Halpern DJ , Crotty K . Low health literacy and health outcomes: an updated systematic review. Ann Intern Med 2011;155(2):97–107. 10.7326/0003-4819-155-2-201107190-00005 21768583

[11] Davis TC , Crouch MA , Wills G , Miller S , Abdehou DM . The gap between patient reading comprehension and the readability of patient education materials. J Fam Pract 1990;31(5):533–8. 2230677

[12] Rudd RE . Improving Americans’ health literacy. N Engl J Med 2010;363(24):2283–5. 10.1056/NEJMp1008755 21142532

[13] Sickle cell disease. Data and statistics. Centers for Disease Control and Prevention. Atlanta, GA. (2011).

[14] Stuart MJ , Nagel RL . Sickle-cell disease. Lancet 2004;364(9442):1343–60. 10.1016/S0140-6736(04)17192-4 15474138

[15] Grosse SD , Schechter MS , Kulkarni R , Lloyd-Puryear MA , Strickland B , Trevathan E . Models of comprehensive multidisciplinary care for individuals in the United States with genetic disorders. Pediatrics 2009;123(1):407–12. 10.1542/peds.2007-2875 19117908

[16] McLaughlin G . SMOG grading: a new readability formula. J Read 1969;(May):639–46.

[17] Mosenthal P , Kirsch I . A new measure for assessing document complexity: the PMOSE/IKIRSCH document readability formula. J Adolesc Adult Literacy 1998;41(8):638–57.

[18] The patient education materials assessment tool (PEMAT) and user’s guide. Rockville (MD): Agency for Healthcare Research and Quality; 2013. http://www.ahrq.gov/professionals/prevention-chronic-care/improve/self-mgmt/pemat/html. Accessed March 1, 2015.

[19] Shoemaker SJ , Wolf MS , Brach C . Development of the patient education materials assessment tool (PEMAT): a new measure of understandability and actionability for print and audiovisual patient information. Patient Educ Couns 2014;96(3):395–403. 10.1016/j.pec.2014.05.027 24973195 PMC5085258

[20] Akter S , Doran F , Avila C , Nancarrow S . A qualitative study of staff perspectives of patient non-attendance in a regional primary healthcare setting. Australas Med J 2014;7(5):218–26. 10.4066/AMJ.2014.2056 24944719 PMC4051357

[21] Clear Communication Index User Guide. Atlanta (GA): Centers for Disease Control and Prevention; 2014.

[22] Guidry JJ , Walker VD . Assessing cultural sensitivity in printed cancer materials. Cancer Pract 1999;7(6):291–6. 10.1046/j.1523-5394.1999.76005.x 10732526

[23] Sickle cell disease. Atlanta (GA): Centers for Disease Control and Prevention. http://www.cdc.gov/ncbddd/sicklecell/freematerials.html. Accessed September 1, 2014.

[24] Explore sickle cell anemia. Bethesda (MD): US Department of Health and Human Services: National Institutes of Health, National Heart, Lung, and Blood Institute; (2012).

[25] American Academy of Family Physicians. Information from your family doctor. When your child has sickle cell disease. Am Fam Physician 2006;74(2):313–4. 16883929

[26] Baur C , Prue C . The CDC Clear Communication Index is a new evidence-based tool to prepare and review health information. Health Promot Pract 2014;15(5):629–37. 10.1177/1524839914538969 24951489

[27] Doak C , Doak L , Root J . Teaching patients with low literacy skills. Philadelphia (PA): J.B. Lippincott Company; 1996.

[28] Hildreth C , Burke A , Glass R . Sickle cell vasculopathy; 2008. http://jama.jamanetwork.com/article.aspx?articleid=183048. Accessed March 1, 2015. 10.1001/jama.300.22.2690 19066390

[29] Salter C , Brainard J , McDaid L , Loke Y . Challenges and opportunities: what can we learn from patients living with chronic musculoskeletal conditions, health professionals and carers about the concept of health literacy using qualitative methods of inquiry? PLoS One 2014;9(11):e112041. 10.1371/journal.pone.0112041 25375767 PMC4222964

[30] Institute of Medicine. Health literacy: a prescription to end confusion. Washington (DC): Institute of Medicine, Committee on Health Literacy; 2004.

